# Perception of learners on the effectiveness and suitability of MyDispense: a virtual pharmacy simulation and its integration in the clinical pharmacy module in Viet Nam

**DOI:** 10.1186/s12909-023-04773-5

**Published:** 2023-10-24

**Authors:** Kim TT Nguyen, My LC Dao, Khoi N Nguyen, Ho N Nguyen, Hoang TM Nguyen, Hoa Q Nguyen

**Affiliations:** 1 Faculty of Pharmacy, University of Medicine and Pharmacy at Ho Chi Minh City, Ho Chi Minh City, Vietnam; 2 School of Pharmacy, Queen’s University Belfast, Belfast, UK

**Keywords:** MyDispense, Simulation programme, Clinical pharmacy

## Abstract

**Background:**

My Dispense is a virtual pharmacy simulation developed for students to train and practice dispensing skills in a safe environment that causes no harm to patients. This study was aimed to investigate learners’ perspectives on the effectiveness of MyDispense and its suitability to integrate into the clinical pharmacy module in Viet Nam.

**Methods:**

A mixed method approach was undertaken. Fourth- and fifth-year pharmacy students at University of Medicine and Pharmacy at Ho Chi Minh city and community pharmacists were invited to complete a survey questionnaire and to participate in semi-structured interviews.

**Results:**

A total of 92/99 participants agreed to take part, of which 75% of participants were students and 65.2% were female. About three-quarters of the participants agreed or strongly agreed that MyDispense improved their dispensing skills, such as patient counselling (70.6%) and collecting patient infomation (85.9%). The majority of the participants (84.8%) considered that MyDispense was suitable to integrate into the clinical pharmacy module. Qualitative analysis from the interviews highlighted the advantages of MyDispense, comprising high interactivity with users, safe environment for practicing medication dispensing, and diversity of common marketed medications. In addition, certain barriers of this programme were also reported, including the complicated process, inconsistent quality of product images, and mixed English-Vietnamese languages.

**Conclusions:**

From learner’s perspectives, MyDispense was an effective tool to enhance dispensing skills and was suitable to integrated into the clinical pharmacy module in Viet Nam.

**Supplementary Information:**

The online version contains supplementary material available at 10.1186/s12909-023-04773-5.

## Background

A pharmacist is required to have strong foundation in pharmaceutical knowledge and skills in dispensing process [1]. This expected learning outcome, perhaps, is one of the biggest challenges to the pharmacy curriculum in Viet Nam. Conventionally, pharmacy students have been provided a series of lectures on medication use to obtain sufficient pharmaceutical knowledge [2]. However, whether skills for pharmacy practice are well trained is uncertain due to limited training hours in the curriculum across universities in Viet Nam [3, 4]. In addition, the internship at a pharmacy only takes place in the final year of the 5-year pharmacy programme and lasts for one week; accordingly, students could hardly conceptualize the pharmaceutical activities and operation procedures at community pharmacies within this short duration [2, 3]. Therefore, a self-learning tool that provides the visualization of pharmaceutical activities in a community pharmacy is necessary, especially when human resources are limited.

In 2011, MyDispense, a web-based pharmacy simulation programme, was first developed by Monash University in Australia [5]. This programme facilitated students with unique in-person experience of dispensing medications to a virtual patient. MyDispense encouraged student-patient communication by some embedded functions in the programme such as patient fact-finding and patient counselling. Notably, MyDispense provided a supportive environment for self-learning in which students could engage in the entire dispensing process, encounter simulated failure, and then receive specific case-by-case feedback from their instructors. By utilising MyDispense, the students were expected to familiarise with common scenarios at a community pharmacy prior entering their internship at a real-world pharmacy.

Since the introduction in 2011, MyDispense has been integrated into the undergraduate pharmacy programmes in Australia, United States of America, United Kingdom, Singapore, Turkey, and Ireland [6]. This integration has received several positive feedbacks. In previous studies, participants found that MyDispense was more interesting than paper-based cases and enhanced their confidence to communicate with patients after completing the provided exercises [7–9]. However, barriers to implementing MyDispense were noted such as some technical issues (e.g. unstable internet access), the cost of maintenance and training, and importantly, the additional workload for both students and lecturers [10, 11]. In Viet Nam, MyDispense was customised for pharmacy students with 30 exercises and 100 medications commonly dispensed in the context of Vietnamese healthcare system. The simulation programme was intended to be blended in the clinical pharmacy modules at University of Medicine and Pharmacy at Ho Chi Minh city (UMP) which were delivered to fourth-year pharmacy students. The customised scenarios were designated to facilitate students’ self-learning with the objectives of mastering pharmacy dispensing knowledge and skills.

This study was aimed to investigate learners’ perspectives on the effectiveness of MyDispense in learning of dispensing skills (patient information collection, prescription review, medication identification, recommendation of over-the-counter (OTC) medications, and patient counselling) and the suitability of this simulation programme to integrate into the clinical pharmacy curriculum in Viet Nam.

## Methods

### Study population

The study inclusion criteria were pharmacists who had been working in community pharmacies or hospital pharmacies for at least one year, and fourth- and fith-year pharmacy students at UMP, Viet Nam from 09/2021 to 07/2022. At the time of conducting this study, fourth- and fith-year pharmacy students had completed their clinical pharmacy modules related to dispensing skills at community pharmacies.

### Sample size

A required sample size was calculated using the following formula where p was the estimated percentage of learners who were satisfied with learning experience using MyDispense:

$$n \ge \frac{{Z}_{1-\frac{\alpha}{2}}^{2}p\left(1-p\right)}{{d}^{2}}$$

$$({Z}_{1-\frac{\alpha}{2}}$$= 1.96 with α = 0.05, d = 0.1)

Based on a study by Johnson et al., p equal to 65.7% was used to calculate the sample size [12]. Thus, the minimum sample size was 87. In this study, invitation emails were sent to 99 participants.

### Study design and data collection

Purposive sampling with the maximum variation approach was used to recruit participants. An invitation was sent to potential participants via email. Pharmacists and pharmacy students volunteering to take part in the study were asked to complete an informed written consent. Subsequently, participants were instructed to use MyDispense via a tutorial video, then required to complete at least one out of five standard dispensing excercises within two weeks prior to entering the study. An automatic reminder email was sent to all participants one week before the deadline. There were no time boundary for each exercise. Participants were allowed to redo five exercises as many times as they wished and also received explanatory notes and feedback immediately after submitting each exercise. All five exercises were developed by the Department of Clinical Pharmacy, UMP, to represent common situations in a community pharmacy in Viet Nam. The five exercises were validated by a committee consisting of 5 senior lecturers and clinical pharmacists.

The original language of command button labels used in MyDispense was translated into Vietnamese to facilitate understanding and the prescription form was modified to align with current regulations in Viet Nam. Images of 100 brand name products marketed in Viet Nam were also added.

Subsequently, a mixed quantitative and qualitative method was undertaken with two phases:

Phase I: An online questionnaire was sent to participants via email (Supplementary 1). All responses were encrypted anonymously. Demographic data including gender and year of study (if participants were pharmacy students) were collected. Participants’ perspectives toward conventional education (the realistic of the traditional paper-based scenarios, the interest of learners toward traditional class, the duration of on-class learning, and their self-confidence in the knowledge and skills gained) were collected using a five-point Likert scale (1 – strongly disagree; 2 – disagree; 3 – neutral; 4 – agree; 5 – strongly agree). The participants were also asked to rate statements about the effectiveness and suitability of MyDispense using the same Likert scale.

Phase II: Participants who completed the questionnaire in phase I were stratified into three groups of fourth year students, fifth year students, and pharmacists. We then selected a random participant from each group to send an invitation email for a face-to-face semi-structured interview. If the recipient refused to take part in the interview, the next random participant in the same group was recruited. The main content of the interview comprised the perspectives of learners regarding the advantages and disadvantages of MyDispense (Supplementary 2). Invitations were sent to participants until data saturation was reached (i.e. no more new perspectives or insights were found in the interview). Each interview was conducted by two authors: M. L-C. D. used a topic guide with predetermined open-ended questions and provided prompts to facilitate discussion while K. T-T. N. observed the interviewee’s reactions and recorded a summary of the interview details. Each interview lasted from 10 to 15 minutes and was audio-recorded.

### Statistical analysis

Data from the online questionnaire were collected, stored, and processed using Microsoft Office Excel and Word 365, and the Statistical Package for Social Sciences (SPSS) version 20.0. Quantitative data such as the categorical variables and Likert scores were presented as frequencies or percentages.

All recordings from the interviews were transcribed verbatim. Data collection and analysis were conducted simultaneously to detect the data saturation point. Transcripts from the interviews along with noted summaries were read iteratively to familiarise context, followed by content analysis to identify themes relevant to MyDispense. Two investigators M. L-C. D. and K. T-T. N. independently analysed the recorded data. If any disagreements between the two investigators could not be addressed through discussion, the final judgment would be made by the third senior investigator (H. Q. N.). The phase II (qualitative approach) was reported according to the Consolidated Criteria for Reporting Qualitative Research (COREQ) guidelines (Supplementary 3).

## Results

### Participants characteristics

Of 99 potential participants approached, 92 (93%) were agreed to take part in the study. Among 92 eligible participants, 65.2% were female. Students accounted for 75% (n = 69) of participants. In phase II, data saturation was reached after 13 participants were interviewed (3 pharmacists and 10 students). Details of participants’ characteristics are presented in Table 1.

**Table 1:** Demographic characteristics of participants

	Phase I (No., %) (n = 92)	Phase II (No., %) (n = 13)
**Occupational status**		
Pharmacy students	69 (75.0)	10 (76.9)
Fourth-year	38 (55.1)	8 (80)
Fifth-year	31 (44.9)	2 (20)
Pharmacists	23 (25.0)	3 (23.1)
**Gender**		
Female	60 (65.2)	8 (61.5)
Male	32 (34.8)	5 (38.5)
**Prior pharmacy experience**		
None	61 (66.3)	5 (38.5)
< 1 year	8 (8.7)	5 (38.5)
1–2 years	10 (10.9)	2 (15.4)
> 2 years	12 (14.1)	1 (7.6)
**Number of exercises completed**		
< 3	38 (41.3)	5 (38.5)
3–5	54 (58.7)	8 (61.5)

### Effectiveness of MyDispense simulation

**Fig. 1:**
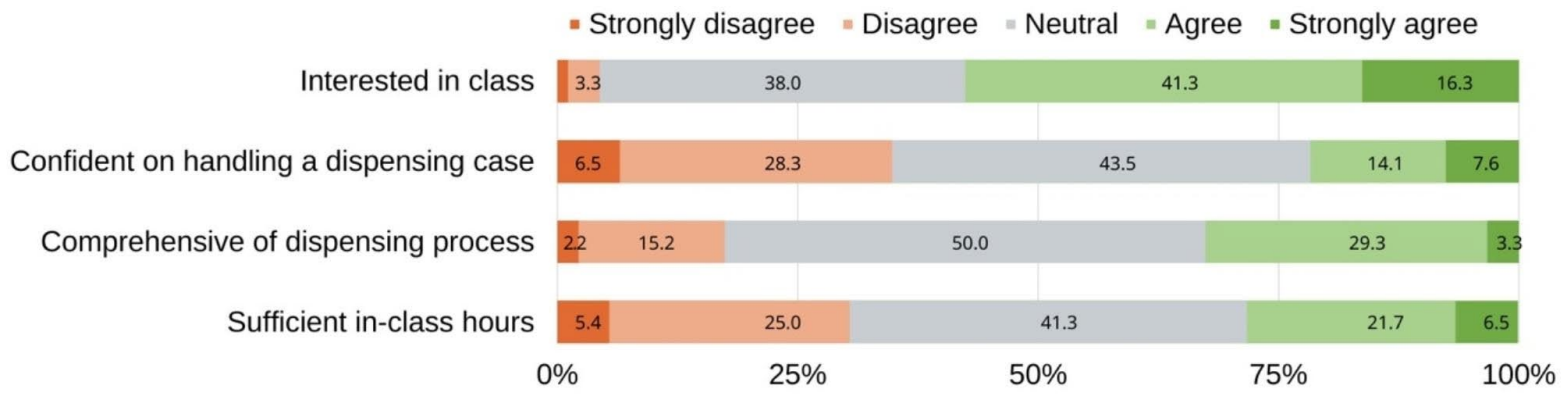
Learners’ perspectives on the traditional teaching approach. The figure demonstrates the percentages of participants agreed/disagreed with the statements on the vertical axis

A total of 56/92 (60.9%) participants supposed that their confidence on handling a dispensing task was improved after using MyDispense compared to 19/92 (21.7%) who also admitted the same effect with the conventional education (Fig. 1; Table 2). The majority of participants (both pharmacists and students) agreed or strongly agreed that MyDispense improved learners’ knowledge and dispensing skills (Table 2). Between pharmacists and students, we found a slightly higher percentage of pharmacists who agreed that MyDispense improved learners dispensing skills including collecting patient medical information, reviewing prescriptions, and recommending OTC medications (Table 3).

**Table 2:** Perception of learners regarding the effectiveness and suitability of MyDispense (N = 92)

	Strongly disagree	Disagree	Neutral	Agree	Strongly agree
*Effectiveness of MyDispense simulation*					
Collecting patient infomation	0 (0)	1(1.1)	12 (13.0)	60 (65.2)	19 (20.7)
Reviewing prescriptions	0 (0)	1(1.1)	14 (15.2)	63 (68.5)	14 (15.2)
Identifying common medicine items	0 (0)	2 (2.2)	22 (23.9)	47 (51.1)	21 (22.8)
Recommending OTC medication	0 (0)	0 (0)	26 (28.3)	59 (64.1)	7 (7.6)
Patient counselling	0 (0)	0 (0)	27 (29.3)	52 (56.5)	13 (14.1)
Confidence on handling a dispensing case	0 (0)	6 (6.5)	30 (32.6)	46 (50)	10 (10.9)
Comprehensive in the dispensing process	0 (0)	2 (2.2)	17 (18.5)	50 (54.3)	23 (25)
Self-learning	0 (0)	0 (0)	13 (14.1)	52 (56.5)	27 (29.3)
*Suitability of MyDispense simulation*					
Suitability to integration into pharmacy course	0 (0)	2 (2.2)	12 (13.0)	48 (52.2)	30 (32.6)
Easy to use	0 (0)	16 (17.4)	28 (30.4)	39 (42.4)	9 (9.8)
Interesting	0 (0)	3 (3.3)	26 (28.3)	47 (51.1)	16 (17.4)
Interactive	0 (0)	3 (3.3)	29 (31.5)	46 (50.0)	14 (15.2)
Realistic	0 (0)	4 (4.3)	22 (23.9)	42 (45.7)	24 (26.1)
Safe practicing environment	0 (0)	4 (4.3)	17 (18.5)	45 (48.9)	26 (28.3)
Matching learning outcomes	0 (0)	1 (1.1)	20 (21.7)	53 (57.6)	18 (19.6)

**Table 3:** Summary of participants in favour of (agreed/ strongly agreed) MyDispense simulation

	Students (N = 69)	Pharmacists (N = 23)
*Effectiveness of MyDispense simulation on improving dispensing skills*		
Collecting patient infomation	59 (85.5)	20 (87.0)
Reviewing prescriptions	57 (82.6)	20 (87.0)
Identifying common medicine items	51 (73.9)	17 (73.9)
Recommending OTC medication	48 (65.2)	18 (73.9)
Counselling	65 (71.0)	16 (69.6)
*Suitability to integrate into pharmacy course*	57 (82.6)	21 (91.3)
Easy to use	37 (53.6)	11 (47.8)
Interesting	48 (69.6)	15 (65.2)
Interactive	44 (63.7)	16 (69.6)
Realistic	49 (71.0)	17 (73.9)
Safe practicing environment	53 (76.8)	18 (78.3)
Matching with learning outcomes	53 (76.8)	18 (78.3)

### Suitability of MyDispense in the clinical pharmacy module

A total of 78/92 participants (84.8%) agreed/strongly agreed that it was necessary to integrate MyDispense into the pharmacy curriculum. Pharmacists and students shared the same perspectives toward the suitability of MyDispense (Table 3). Majority of the participants (71.8%) believed that the scenarios in MyDispense were realistic, interesting (68.5%), interactive (65.2%) and strongly connected to the intended learning objectives (77.2%). However, 17.4% of participants disagreed that MyDispense was a user-friendly programme (Table 2).

### Learner’s perspectives on MyDispense simulation

After 13 interviews, three main themes regarding the advantages of MyDispense were identified (Table 4). Firstly, eight participants agreed that this simulation was advanced with an interactive platform. In particular, the design of the virtual pharmacy was elegant and perfectly replicated a real modern pharmacy. Virtual patients were designed to mimic the characteristic of real patients including their emotions such as anxiety, anger, or impatience. One participant also reported that learners could gather sufficient patient medical information by the “fact-finding” function. One participant enjoyed the accessibility of MyDispense on different mobile devices including smartphones.

**Table 4:** The advantages and disadvantages of MyDispense from learners’ perspectives

Advantages/Disadvantages	Quotes
**Advantages**	
High interactivity with users (8 participants) • Standard virtual pharmacy • Lively virtual patients • Accessible from several devices	“I could observe patient appearance including their ages, genders and other special features such as pregnant women, so it helps me visualize better” (ST_F_6) “I liked that MyDispense can be used in my phone so I can do it anywhere when I have time” (PH_F_3)
Safe environment for practicing (6 participants) • Productive failure • Harmless to real patients	“One function that I find very cool is the feedback, which helps me have the ability to self-study and self-check whether the prescription I give to the patient is correct or not” (ST_F_5)
Diversity of common marketed medications (7 participants)	“…it helps me get used to some brand names, because it’s less common when I’m studying…” (ST_M_7).
**Disadvantages**	
Complicated learning process (9 participants)	“…it took me a lot of time to find the right medication on the shelves” (ST_M_7).
Inconsistent quality of product images (9 participants)	“Some medication labels were very hard to read” (PH_F_1)
Mixed languages (8 participants)	“It would be better that all excercises would be written in Vietnamese” (PH_F_1)

Secondly, almost half of the participants remarked that MyDispense offered a safe practicing environment for learners with no prior experience. Some participants also appreciated that they could redo each exercise as many times as they wished, which was helpful to reduce potential mistakes regarding patient safety.

Finally, more than half of the participants indicated that MyDispense possessed a diversity of commonly marketed medications. Three participants revealed that they could easily familiarise themselves with some common brand name products through the provided scenarios before entering the pharmaceutical market (Table 4).

Nevertheless, some of the barriers were also mentioned by most of the participants, including complicated learning process (time consuming to get used to the programme), inconsistent quality of product images (unreadable medication labels), and mixed languages (Vietnamese and English).

## Discussion

To our knowledge, this study is the first one that examined the perception of Vietnamese learners toward a virtual pharmacy simulation programme. Similarly to previous findings from other countries [7–9], the majority of participants, including pharmacy students and pharmacists, agreed that MyDispense was an effective tool to train dispensing skills and should be integrated into the pharmacy curriculum in Viet Nam.

MyDispense enhanced learners’ dispensing skills and supported self-learning. After using this simulation programme, the proportion of participants who were confident to handle a dispensing task increased from 21.7 to 60.9%. These findings were consistent with previous studies in which 71-75.4% of users agreed/strongly agreed that MyDispense improved their knowledge and dispensing skills [12–14]. Simulation activities have been reported to promote better knowledge retention and performance of clinical skills [15, 16]. Moreover, it should also be noted that the pharmacists participating in our study favoured how MyDispense improved learner’s ability to collect patient medical information, review prescriptions, and recommend OTC medications, while the pharmacy students preferred how the simulated scenarios enhanced their counselling skills (Table 3).

Majority of participants believed MyDispense should be integrated into the pharmacy curriculum. These findings were consistent with previous studies conducted in the United States [13, 14]. Nevertheless, a study by Shin et al. reported only 20.7% of users agreed to integrate MyDispense into their curriculum [8]. This discrepancy may be explained by the lack of sufficient training prior to the use of MyDispense as presented in the study. It should be mentioned that pharmacists were also recruited in our study. We found that the proportion of students who agreed/strongly agreed to integrate this simulation to their course was lower than that of pharmacists (82.6% vs. 91.3%). This is possibly because the pharmacists expected what they should have been trained for their current positions at their pharmacies, and the students might be concerned about the pressure of additional homework and classwork tasks.

Integrating MyDispense into the clinical pharmacy curriculum will probably tackle current challenge of the traditional teaching method by optimising students learning experience. In our current pharmacy curriculum, we did not provide any specific tool for students to conceptualize their professional activities or for self learning at home [2]. Most of our participants agreed/strongly agreed that the feedback function of MyDispense encouraged their self-learning compared to the traditional teaching method. A previous study indicated that MyDispense supported students to learn from their own experience [16]. Additionally, the participants reported that MyDispense provided more realistic experience than the conventional education with paper scenarios, which was also highlighted in previous studies [16, 17].

In the semi-structured interviews, the participants described three main advantages of MyDispense: high interactivity with users, provision of safe environment for dispensing practice, and the diversity of common marketed medications. This could be due to the fact that MyDispense was the first simulation programme adapted for Vietnamese users. To date, the current education approach in Viet Nam has not provided enough individual self-training before the pharmacy internship period. Moreover, pharmacy students were more familiar with names of active ingredients through class lectures than brand names of medications; therefore, three-dimension images of medication packaging embedded in MyDispense could enhance students’ ability to recognise common brand-name products which they may encounter during their pharmacy internship or after graduation.

Nevertheless, the participants also outlined certain barriers when using MyDispense, comprising complicated learning process, inconsistent quality of medication images, and mixed languages. A previous study also reported that students in Philippines encountered connectivity issues and gadget incompatibility when using MyDispense [18]. This discrepancy may be due to different adoptation process in our study. The participants were only instructed to use MyDispense with a short video instead of in-person lecturing which was undertaken in previous studies [16, 17]. By the time of the study conducted, the version of MyDispense was originally set English as default language instead of Vietnamese [6]. However, these problems could be addressed in the future with further improvement and adaptation of MyDispense.

While the results are in favor of integrating MyDispense into the pharmacy curriculum, they should be carefully interpreted. Firstly, the findings are vulnerable to bias resulted from a considerable proportion of participants (41.3%) completed less than 3 out 5 excercises. Secondly, in this present study, the effectiveness of MyDispense was evaluated according to learners’ opinions which could be subjective. Moreover, the endowment effect may have occurred as participants were allowed to redo the exercises as many times as they wished. Nevertheless, we accepted this practice as the participants probably took time to familiarise themselves with MyDispense which had first been developed in the non-Vietnamese context despite our adaption. Further studies are warranted to re-assess the effectiveness of MyDispense by objective measures such as exam scores before and after using this novel virtual programme.

## Conclusions

From learners’ perspectives, MyDispense effectively improved their dispensing skills and was suitable for the integration to the Vietnamese pharmacy curriculum. Further investigation is warranted to assess feasibility and acceptability after MyDispense is integrated into the pharmacy education curriculum in Viet Nam.

### Electronic supplementary material

Below is the link to the electronic supplementary material.

Supplementary Material 1

## Data Availability

The datasets used and/or analysed during the current study are available from the corresponding author on reasonable request.
