# Supplementary material for: Perception of learners on the effectiveness and suitability of MyDispense: a virtual pharmacy simulation and its integration in the clinical pharmacy module in Viet Nam
Source: BMC Med Educ. 2023 Oct 24;23:790. doi: 10.1186/s12909-023-04773-5 (PMC10599015; doi:10.1186/s12909-023-04773-5)
Supplement: Supplementary file 1 — Supplementary Material 1 [file 12909_2023_4773_MOESM1_ESM.docx]

Perception of learners on the effectiveness and suitability of MyDispense: a virtual pharmacy simulation and its integration in the clinical pharmacy module in Viet Nam

**Authors:** Kim TT Nguyen,^1^ My LC Dao,^1^ Khoi N Nguyen,^1^ Ho N Nguyen,^1^ Hoang TM Nguyen,^1^ Hoa Q Nguyen. ^1.2*^

^1^Faculty of Pharmacy, University of Medicine and Pharmacy at Ho Chi Minh City, Ho Chi Minh City, Viet Nam.

^2^School of Pharmacy, Queen’s University Belfast, Belfast, United Kingdom.

*Corresponding author:*

Hoa Q Nguyen, PhD. Email: nqhoa@ump.edu.vn; h.q.nguyen@qub.ac.uk

**Supplementary 1.** Questionaire in phase I to investigate the perception of learners toward MyDispense)

*Part 1. Participant’ perspectives toward conventional education*

| No. | Question | Rating |
| --- | --- | --- |
| 1 | The traditional paper-based scenarios that you have learned were realistic. | 1- Strongly disagree  2- Disagree  3- Neutral  4- Agree  5- Strongly agree |
| 2 | The traditional class was interesting. |  |
| 3 | The duration of traditional class was sufficient. |  |
| 4 | You are confident to dispense medications to a real patient in a pharmacy after finishing your course |  |

*Part 2. Participant’ perspectives toward MyDispense*

| No. | Question | Rating |
| --- | --- | --- |
| The effectiveness of MyDispense | | |
| 1 | MyDispense improved learner’s skill for collecting patient medical information. | 1- Strongly disagree  2- Disagree  3- Neutral  4- Agree  5- Strongly agree |
| 2 | MyDispense improved learner’s skill for reviewing prescriptions. |  |
| 3 | MyDispense improved learner’s skill for identifying common medicine items. |  |
| 4 | MyDispense improved learner’s skill for selecting OTC medications. |  |
| 5 | MyDispense improved learner’s skill for counselling. |  |
| 6 | MyDispense improved learner’s knowledge regarding the dispensing process. |  |
| 7 | MyDispense improved self-study by giving immediate feedbacks. |  |
| 6 | You are confident to dispense medications to a real patients in a pharmacy after self-study with MyDispense.* |  |
| The suitability of MyDispense to integrate into the clinical pharmacy module | | |
| 7 | MyDispense simulated scenarios are fit with learning objectives | 1- Strongly disagree  2- Disagree  3- Neutral  4- Agree  5- Strongly agree |
| 8 | MyDispense simulated scenarios are realistic |  |
| 9 | MyDispense provided learners with a safe environment for practicing |  |
| 10 | MyDispense promoted learners-patients communication |  |
| 11 | MyDispense is easy to use |  |
| 12 | Self-study with MyDispense is interesting |  |
| 13 | It is necessary to integrate MyDispense into  clinical pharmacy practice 1 module. |  |

** applied to pharmacy students only*

**Supplementary 2.** Prepared questions for semi-structured interviews

| No. | Question |
| --- | --- |
| 1 | How do you feel after using MyDispense? |
| 2 | What are the advantages of MyDispense? |
| 3 | What is your favorite function of MyDispense, and why? |
| 5 | What are the disadvantages of MyDispense? |
| 6 | In your opinion, what functions should be added to improve user’s experience? |

**Supplementary 3.** Consolidated criteria for reporting qualitative studies (COREQ): 32-item checklist

| **No. Item** | **Guide questions/description** | **Reported** |
| --- | --- | --- |
| **Domain 1: Research team and reﬂexivity** | | |
| *Personal Characteristics* | | |
| 1. Inter viewer/facilitator | Which author/s conducted the interview or focus group? | M L-C D, K T-T N |
| 2. Credentials | What were the researcher’s credentials? E.g. PhD, MD | M L-C D Bsc  K T-T N, H TM N Msc  H Q. N PhD |
| 3. Occupation | What was their occupation at the time of the study? | M L-C D: Student  K T-T N, H TM N, H Q. N: Lecturers |
| 4. Gender | Was the researcher male or female? | M L-C D, K T-T N, H TM N: female  H Q. N: male |
| 5. Experience and training | What experience or training did the researcher have? |  |
| *Relationship with participants* | | |
| 6. Relationship established | Was a relationship established prior to study commencement? | Yes |
| 7. Participant knowledge of the interviewer | What did the participants know about the researcher? e.g. personal goals, reasons for doing the research | Participants were briefed about the objectives of this study, understood it and gave written informed consent. |
| 8. Interviewer characteristics | What characteristics were reported about the interviewer/facilitator? e.g. Bias, assumptions, reasons and interests in the research topic | Each interview was conducted by 2 researcher to minimise bias |

| **Domain 2: study design** | | |
| --- | --- | --- |
| *Theoretical framework* | | |
| 9. Methodological orientation and Theory | What methodological orientation was stated to underpin the study? e.g. grounded theory, discourse analysis, ethnography, phenomenology, content analysis | Theme analysis |
| *Participant selection* | | |
| 10. Sampling | How were participants selected? e.g. purposive, convenience, consecutive, snowball | Purposive sampling |
| 11. Method of approach | How were participants approached? e.g. face-to-face, telephone, mail, email | Email |
| 12. Sample size | How many participants were in the study? | 13 |
| 13. Non-participation | How many people refused to participate or dropped out? Reasons? | None |
| *Setting* | | |
| 14. Setting of data collection | Where was the data collected? e.g. home, clinic, workplace | A classroom in the university |
| 15. Presence of non-participants | Was anyone else present besides the participants and researchers? | No |
| 16. Description of sample | What are the important characteristics of the sample? e.g. demographic data, date | Their occupation (students and pharmacists) |
| *Data collection* | | |
| 17. Interview guide | Were questions, prompts, guides provided by the authors? Was it pilot tested? | Yes |
| 18. Repeat interviews | Were repeat inter views carried out? If yes, how many? | No |
| 19. Audio/visual recording | Did the research use audio or visual recording to collect the data? | Audio record |
| 20. Field notes | Were ﬁeld notes made during and/or after the interview or focus group? | Notes were made during the interview |
| 21. Duration | What was the duration of the inter views or focus group? | 10-15 mins |
| 22. Data saturation | Was data saturation discussed? | Yes |
| 23. Transcripts returned | Were transcripts returned to participants for comment and/or correction? | No |
| **Domain 3: analysis and ﬁndings** | | |
| *Data analysis* | | |
| 24. Number of data coders | How many data coders coded the data? | One (M L-C D) |
| 25. Description of the coding tree | Did authors provide a description of the coding tree? | No |
| 26. Derivation of themes | Were themes identiﬁed in advance or derived from the data? | Themes were derived from the data |
| 27. Software | What software, if applicable, was used to manage the data? | Microsoft Word and Excel |
| 28. Participant checking | Did participants provide feedback on the ﬁndings? | No |
| *Reporting* | | |
| 29. Quotations presented | Were participant quotations presented to illustrate the themes/ﬁndings? Was each quotation identiﬁed? e.g. participant number | No |
| 30. Data and ﬁndings consistent | Was there consistency between the data presented and the ﬁndings? | Yes |
| 31. Clarity of major themes | Were major themes clearly presented in the ﬁndings? | Yes |
| 32. Clarity of minor themes | Is there a description of diverse cases or discussion of minor themes? | Yes |
